# What features do Dutch university students prefer in a smartphone application for promotion of physical activity? A qualitative approach

**DOI:** 10.1186/s12966-015-0189-1

**Published:** 2015-03-01

**Authors:** Anouk Middelweerd, Danielle M van der Laan, Maartje M van Stralen, Julia S Mollee, Mirjam Stuij, Saskia J te Velde, Johannes Brug

**Affiliations:** Department of Epidemiology & Biostatistics and the EMGO Institute for Health and Care Research, VU University Medical Center, Amsterdam, the Netherlands; Department of Earth and Life Science and the EMGO Institute for Health and Care Research, VU University Amsterdam, Amsterdam, The Netherlands; Department of Computer Science, VU University Amsterdam, Amsterdam, The Netherlands; Department of Medical Humanities and the EMGO Institute for Health and Care Research, VU University Medical Center, Amsterdam, the Netherlands; Mulier Institute, Utrecht, the Netherlands

## Abstract

**Background:**

The transition from adolescence to early adulthood is a critical period in which there is a decline in physical activity (PA). College and university students make up a large segment of this age group. Smartphones may be used to promote and support PA. The purpose of this qualitative study was to explore Dutch students’ preferences regarding a PA application (PA app) for smartphones.

**Methods:**

Thirty Dutch students (aged 18–25 years) used a PA app for three weeks and subsequently attended a focus group discussion (k = 5). To streamline the discussion, a discussion guide was developed covering seven main topics, including general app usage, usage and appreciation of the PA app, appreciation of and preferences for its features and the sharing of PA accomplishments through social media. The discussions were audio and video recorded, transcribed and analysed according to conventional content analysis.

**Results:**

The participants, aged 21 ± 2 years, were primarily female (67%). Several themes emerged: app usage, technical aspects, PA assessment, coaching aspects and sharing through social media. Participants most often used social networking apps (e.g., Facebook or Twitter), communication apps (e.g., WhatsApp) and content apps (e.g., news reports or weather forecasts). They preferred a simple and structured layout without unnecessary features. Ideally, the PA app should enable users to tailor it to their personal preferences by including the ability to hide features. Participants preferred a companion website for detailed information about their accomplishments and progress, and they liked tracking their workout using GPS. They preferred PA apps that coached and motivated them and provided tailored feedback toward personally set goals. They appreciated PA apps that enabled competition with friends by ranking or earning rewards, but only if the reward system was transparent. They were not willing to share their regular PA accomplishments through social media unless they were exceptionally positive.

**Conclusions:**

Participants prefer PA apps that coach and motivate them, that provide tailored feedback toward personally set goals and that allow competition with friends.

## Background

The positive effects of regular physical activity (PA) are well known, yet many people do not comply with PA guidelines [1,2]. Sixty-four percent of Dutch young adults (aged 18–34 years) meet the guidelines for being physically active at a moderate intensity for at least 30 minutes per day and at least 5 days per week [3]. The transition from adolescence to adulthood and the period of early adulthood itself is a critical period during the life course where the decline of PA appears to accelerate [4,5]. Previous studies indicate that the rate at which PA decreases varies between men and women, and men who transition into a university are more likely to adopt a less physically-active lifestyle [4]. In the Netherlands, many students who enter university move away from home, start to live on their own or in student housing communities and combine their study obligations with part-time jobs and social commitments. This may result in a reduction in free time that was previously available for PAs [6].

Smartphones and smartphone applications (apps) are popular, especially among highly educated young adults [7] and offer new possibilities for promoting PA. The rapidly growing number of fitness apps that are commercially available indicate their popularity [8]. However, recent studies and a systematic review show that most of them are minimally based on established behaviour change theories and techniques [9-12]. The review by Middelweerd et al. [12] further demonstrates that when established behaviour change techniques are included, self-monitoring (e.g., GPS, diary, or accelerometer), goal-setting features and social support by connecting with social networking sites (e.g., Facebook or Twitter) were applied most frequently.

A small number of studies examine the usability and effectiveness of PA apps to increase PA in healthy (young) adults [13-17]. Glynn et al. [14] report significant increases (1029 steps) in daily step activity in the intervention group using an app that offered feedback graphs and continuous feedback. Kirwan et al. [13] conclude that a smartphone app in addition to website-delivered intervention could enhance engagement and increase levels of PA. Thus far, PA app interventions are commonly used as supplemental tools, complementing the primary goal of keeping track of personal goals [13,16], making ecological momentary assessments [17] or providing feedback for current behaviour [18]. Yet, little is known about the preferences of young adults for specific behaviour change techniques applied in a PA app that stands on its own.

Social networking sites such as Facebook and Twitter are popular among Dutch young adults: 98 percent use Facebook and/or Twitter [7]. Many PA apps offer the possibility of sharing one’s activities through social media. However, little is known about whether Dutch students like the possibility of sharing PA-app-based tracking of their activities through social media and if they actually share their results.

Developing a theory-based, effective and engaging PA app that is also based on user preferences and opinions is a complex process, as are all thoroughly-developed theory- and evidence-based interventions [19]. A recent review identifies key features that facilitate PA engagement: real-time feedback, social networking, expert consultations and goal setting. In addition, disruptive prompts, text messaging and competition-based strategies reportedly limit engagement in PA [20]. However, little is known about the usage, appreciation and preferences of students (aged 18–25 years) for various features in such apps. Understanding their needs, expectations and preferences is the first step in designing more effective PA apps.

This study aimed to gain insight into the role, usability and appreciation of an existing PA app that allows sharing of activities through social media, called *Nexercise* [21]. Three research questions were addressed: (1) How do Dutch bachelor’s and master’s students (aged 18–25 years) use and appreciate the various features of an existing PA app? (2) What are the preferences of Dutch bachelor’s and master’s students regarding a new PA app? (3) How do Dutch bachelor’s and master’s students use and appreciate the option to share accomplishments through social media?

## Methods

### Design

A qualitative design was used to explore respondents’ preferences, attitudes and experiences regarding PA apps; for this reason, focus group discussions were the chosen format [22]. To ensure meaningful focus group discussions, participants must have had some experience with a PA app. They were asked to download the *Nexercis*e app (version 2.2.3; www.nexercise.com) [21] and then use it during the three weeks preceding the discussions. The *Nexercise* app is a GPS fitness tracker that can be used for a variety of sports activities such as fitness, running and, horseback riding, and contains multiple options such as GPS tracking, activity log book, earning points, a competition feature, chat features and linking with social media. This PA app was selected because (1) it was found to include behaviour change theories and techniques in a recent review, such as prompting goal setting, prompting self-monitoring, providing feedback on performance, providing rewards and planning social support [12]; (2) it was freely available from both iTunes and Google Play and thus was compatible with both iPhones and Android smartphones; (3) it enabled tracking a variety of PA behaviours, so it was not focused on only one sport or activity; and (4) the app consisted of multiple features, including GPS tracking, rewarding, ranking, chat and the possibility of sharing results. Participants were asked to use the app when engaging in PA and to post their accomplishments on their social media pages. Use of the app and sharing was completely voluntary, and participants were informed that they could participate in the focus group discussions whether or not they used the app.

The study was approved by the Medical Ethical Committee of the VU Medical Centre, Amsterdam.

### Recruitment

This study was conducted using Dutch bachelor’s and master’s students at the VU University, Amsterdam between April and June 2013. Eligibility required the participants be current students (bachelor’s or master’s) aged between 18 and 25 years, healthy and without contraindications for sports participation, own a smartphone with internet access, be a member of Facebook or Twitter, and have mastery of the Dutch language. The recruitment took place in person by distributing flyers and through online social media advertisements, and eligible persons were informed that they could receive an incentive for their participation (i.e., an arm holder for a smartphone and voucher for free entrance to the university sports centre). An effort was made to include participants who were at various PA levels because this might affect their preferences for specific features of a PA app. Participants were divided based on whether or not they met the Dutch PA guidelines. The PA levels were assessed using the Dutch short version of the International Physical Activity Questionnaire (IPAQ) [23]. Participants were considered to meet the Dutch PA guidelines if they reported at least 30 minutes of moderately intense PA daily for at least five days per week or at least 20 minutes of vigorous activity daily for at least three days per week [3]. An effort was made to create homogenous focus groups based on the participants’ PA levels according to the IPAQ, resulting in three groups comprising participants who met the guidelines and two groups comprising participants who did not meet the guidelines.

### Procedures

To streamline the focus group discussions, a discussion guide was developed which included open-ended questions and prompts (statements) to encourage participants to share their opinions. The prompts aimed to provoke discussion about topics that were not yet covered and were used at the end of each discussion. Three prompts were used: 1) “I enjoy using a smartphone app during my sports activities, but only a couple of times. After awhile I do not use the app anymore.” 2) “Positive feedback on my physical activity achievements from my friends encourages me to be more physically active.” 3) “It really annoys me when my friends on Facebook post their sports activities on their timeline.” Table 1 provides an overview of the topics included in the discussion guide. An example of an open-ended question is, “When do you usually use the app and for what kinds of activities?”

**Table 1:** **Main topics of the focus group discussion guide**

**Number**	**Topics**
1	General smartphone application usage
2	General impression of *Nexercise* ^a^
3	Usage and appreciation of *Nexercise* ^a^
4	Usage and appreciation of various features
5	Preferences for various features
6	Social support through an application
7	Sharing through social media (e.g., Facebook or Twitter)

^a^Nexercise = fun and weight loss: physical activity smartphone application for iOS and Android.

To obtain demographic characteristics, participants were asked to complete a short online questionnaire prior to the focus group discussion. The first page of this questionnaire contained information about the study and included an informed consent for the questionnaire, ensuring anonymity and confidentiality, and which required the participant’s signature before the remainder of the questionnaire could be completed.

Written informed consents for the focus group discussions were obtained prior to the discussions, which spanned one hour each and were led by a trained moderator (DMvdL) who was an age peer of the participants. Prior to the first focus group discussion, the moderator attended a workshop on qualitative research and pilot-tested the discussion guide under the supervision of a qualitative research expert (MS). During the discussions, the moderator assured that participants were aware of the purpose and procedures, noted that they were audio and video recorded and ensured confidentiality and anonymous transcriptions. Two additional researchers (TV and AM, TV and MMvS or TV and JSM) assisted with the discussions by acting as practical assistants and observers and took the opportunity to ask the participants questions, clarifying any remaining concerns at the end of the discussions. At the close of each discussion, participants were given forms thanking them for participating and asking for written comments and then were awarded with the incentive. The comments could include issues they wanted to share but did not mention during the discussion and any comments regarding the topics that were discussed or topics they thought should have been discussed. The members of the research team attending the discussion evaluated it by sharing first impressions and assessing the role of the moderator.

### Data management and analysis

The recordings were transcribed verbatim (DMvdL) with pseudonyms for each respondent. The transcripts were checked for quality and completeness by another researcher (TV) and were analysed according to conventional content analysis, generally used when little research has been done in the subject area and little is known [22]. Atlas.ti 6.0, software for qualitative analysis, was used to perform the analyses. First, the transcripts were read verbatim independently by two researchers (DMvdL and TV) to select relevant fragments based on the discussion guide. Various codes and sub codes were created with these fragments. Second, the codes were reviewed and split, combined, added or removed (DMvdL) if overlapping codes or better coding names were discovered. Third, the codes and subcodes were clustered and sorted into general themes (DMvdL), and a tree diagram was composed to provide a visual representation of the codes. Several meetings of the research team (TV, SJtV, MMvS, JSM, MS) were arranged so that consensus could be reached. All fragments were split according to the focus group discussion, which implies splitting data based on PA level (whether or not the participants met PA guidelines). This was done with the aim of finding remarkable differences between the groups. Once these differences were found and described, the data were combined for analysis as one dataset.

## Results

### General characteristics

**Figure 1:**
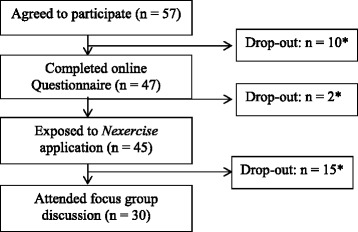
**Flowchart of the study procedure, drop-out rates and reasons.**

Fifty-seven participants agreed to participate, yet 30 (53%) attended the focus group discussions. Figure 1 shows a flow chart of participant dropouts and reasons.

The participants (n = 30) were aged 21 ± 2 years and were primarily female (67%) living in Amsterdam (50%) and did not meet PA guidelines (57%). The focus groups ranged from 4 to 7 members each. Participant characteristics are listed in Table 2. Within the groups comprising participants with higher PA levels, four participants (two male) were very active, reporting vigorously activity for at least 20 minutes 5 to 9 times weekly.

**Table 2:** **Focus group characteristics per focus group discussion and for participants who dropped out of the study**

**Focus group**	**Number of participants**	**Sex (number of females)**	**NNGB** ^**1**^ **(number meeting the guidelines)**	**Fit norm** ^**2**^ **(number meeting the guidelines)**	**Number meeting the guidelines (NNGB and/or Fit norm)** ^**3**^	**Sports**
1	7	4	6	6	6	Gym workout (n = 5)
						Cycling (n = 3)
						Climbing (n = 1)
						Inline skating (n = 1)
						Pool dancing (n = 1)
						Physiotherapy exercises^5^ (n = 1)
2	7	2	6	7	5	Running (n = 4)
						Ice skating (n = 2)
						Field hockey (n = 1)
						Swimming (n = 1)
						Cycling (n = 2)
						Spinning (n = 1)
						Gym workout (n = 3)
						Climbing (n = 1)
						Indoor soccer (n = 1)
3	5	5	2	0	0	No sports (n = 2)
						Running (n = 2)
						Gym workout (n = 1)
4	4	2	1	0	1	Running (n = 3)
						Gym workout (n = 2)
						Mountain bike (n = 1)
						Surfing (n = 1)
						Inline skating (n = 1)
						Spinning (n = 1)
5	7	7	1	0	1	No sports (n = 3)
						Running (n = 2)
						Gym workout (n = 1)
						Handball (n = 1)
						Field Hockey (n = 1)
						Spinning (n = 1)
						Box training (n = 1)
Drop outs^4^	27	14	18	16	15	No sports (n = 4)
						Gym workout (n = 7)
						Running (n = 4)
						Soccer (n = 3)
						Field hockey (n = 2)
						Cycling (n = 1)
						Water polo (n = 1)
						Basketball (n = 1)
						Badminton (n = 1)
						Pool dancing (n = 1)

^1^NNGB = Dutch health guidelines for physical activity (30 minutes of moderate activity at least 5 days per week).

^2^Fit norm = 20 minutes of vigorous activity at least 3 days per week.

^3^The number of participants who met the Dutch health guidelines for physical activity (NNGB) and/or the Fit norm.

^4^Number of participants who dropped out during the study.

^5^Engage in a fitness programme that is provided and supervised by a physiotherapist.

### General themes

**Figure 2:**
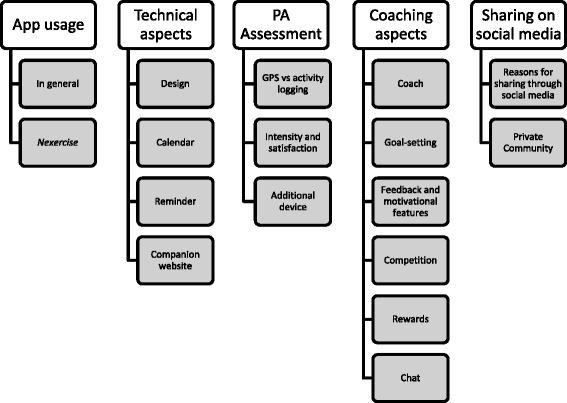
**Overview of the themes and sub-themes discussed in all focus groups.**

Five general themes emerged in all focus group discussions: general app usage, technical aspects, PA assessment, coaching aspects and sharing through social media. Figure 2 provides an overview of the themes and subthemes. In general, the same topics were discussed, and similar themes emerged in all groups.

### App usage

#### App use in general

The types of smartphone apps most often used by the participants were social networking apps such as Facebook and Twitter, communication apps (e.g., WhatsApp) and content apps (e.g., news reports, weather forecasts or public transport information). Game apps were not very popular; respondents stated that such apps were amusing or pleasant for short-term use only. Some participants, primarily those meeting the PA guidelines, already used a comparable PA app such as RunKeeper, Endomondo or Strava.

### Use of the *Nexercise* app

In all focus group discussions except that of Group 4, the participants had a positive attitude toward PA apps. Group 4 comprised participants with lower levels of PA, and these participants stated they did not need such an app. Participants with higher PA levels clearly believed that the *Nexercise* app would be useful for inactive people, to raise awareness that they need to be more physically active, for example. However, they perceived the *Nexercise* app to be less useful for themselves. Self-reported app use revealed that all participants used the *Nexercise* app at least once with one exception. On average, it was used 8 times (range 0–29).

The frequency of *Nexercise* app use varied among participants. For some, it became routine to start the tracking feature when they intended to exercise. *“When the app was still working on my phone, it became natural to use the app” (male who did not meet PA guidelines). “For me the app usage decreased over time. I used to be a fanatical user by always starting the app, but after a while I couldn’t care anymore” (male who did not meet PA guidelines).* For others, it was something they would easily forget. Often, the preceding mind-set appeared to determine the frequency of use (e.g., participants who found the app unnecessary, time consuming and not useful beforehand did not use it often or at all). Some participants mentioned that they exercised only for themselves and they did not require the support of a smartphone app.

The majority of the participants reported becoming more aware of their PA level, such as their activity patterns and duration of their activities. They also saw that short distances could make a difference. *“I became more aware of the fact that I am actually pretty active. I thought that I was doing nothing, but afterwards I was not that bad as I thought I was” (female who did not meet PA guidelines).*

During all focus group discussions, the participants were presented with the following statement at the end of the discussion: “I enjoy using a smartphone app for a short period, but after that I do not use the app anymore.” Almost all participants agreed with this statement, saying they experienced this feeling with almost all smartphone apps they used. At some point, the novelty disappeared, and when they experienced a problem with the app, such as a stuttering mobile phone, lack of storage or battery problems, they often stopped using it. *“It does not personally add anything for me… so you will quit using it, you can see it as useless and something you want to get rid of” (male who did not meet PA guidelines).* Participants mentioned that if the app was more tailored to their needs and if they gained added value from it, they would probably still use it.

### Technical aspects

#### Design

In general, the participants preferred to have a simple and well-ordered app design. They wanted to have a structured layout with only a few important features which could easily and effortlessly log activities and obtain a clear overview of the results. Some participants wanted to customise it themselves. *“Maybe you could adapt the starting page of the application and choose a quick forward button, so you could easily go to the option you prefer… with the possibility to add or remove additional options (male who did not meet PA guidelines)*.

Most participants appreciated the enormous list of activities included in the *Nexercise* app, but they found the list to be rather limited for some activities, such as fitness trainings.

### Calendar

Participants with higher levels of PA liked the idea of a calendar within the app, providing them with an overview of their accomplishments. Some participants did not need such a schedule because they could use their own agenda. *“I used it most often to log afterwards,… like trips to school and stuff like that…”(male who met PA guidelines). “What I like as well is the calendar…. I don’t work out on a regular basis, so if you look back you have an overview on which days you did what kind of sports…”(male who met PA guidelines). “But I usually write it down in my regular agenda, thus it is twice as much work to keep that diary as well” (female who met PA guidelines). “Those agendas should be merged” (male who did not meet PA guidelines). “Yes” (female who met PA guidelines*). The participants who did not meet guidelines thought of the app in terms of replacement for a coach telling them what to do. Thus, they wanted the calendar function to make a training schedule and to set tasks for them.

### Reminders

Most of the participants who had lower levels of PA perceived the reminders as annoying. One reason for this was the feeling that they were able to decide for themselves when they wanted to exercise. Another reason was the potential feeling of guilt that did not work for them; a third was that they did not want to be bothered with notifications reminding them to exercise. However, participants with higher levels of PA more often appreciated the reminders, although they also highlighted that they did not always come at appropriate moments. For instance, they came after recent PA or when it was late in the evening. Some participants reported that the reminders were tools that triggered them to make time to exercise or to fill out more activity results. “*For me it was more like a reminder,… I need to fill in my diary today,… that I really got the feeling I need to work out tonight” (male who met PA guidelines).*

### Companion website

Almost all participants preferred having an account on a website in addition to the app. Reasons for this were that they could add data more easily and that it could present much more information about their activities, such as progress bars, activity schedules, graphics, maps and routes. *“…that it finds a route…; I want 5 km and then the website tells you which route” (male who did not meet PA guidelines).* Instructional videos, tips and forums came up in the discussions only among those with lower levels of PA. They indicated that such a website should be an additional support system where they could access more detailed and in-depth information and tips on how to perform a workout. In contrast, the participants with higher levels of PA preferred a website that presented additional and more detailed information about their workout (e.g., average speed and heart rate) and their progress. A few participants noted that such a website could be a barrier for use of the app. *“Yes, an additional website for support with the option to set a goal and to reach it. But it is an additional barrier to go and visit the website” (female who did not meet PA guidelines).*

### Physical activity assessment

#### GPS tracking vs. activity logging

*Nexercise* provided various options for logging activities. Some participants consistently logged their activities after exercise because they knew the exact information they needed or because it was not possible to track their activities with GPS. Examples of such activities are swimming or playing soccer. *“Actually, I only logged my activity afterwards and once I took it for a run, but for everything else like spinning I wouldn’t take it with me” (male who met PA guidelines)*. Additionally, some participants admitted that they often forgot to start the tracking feature, so they could log recent activities only. For others, it became routine to log activities at the end of the day. A small number found it very annoying to carry their smartphones with them during exercise. It was often mentioned that because tracking with GPS consumes battery power, participants felt they had no choice and would log activities only after exercise. Some found it unnecessary to log activities because they had already completed their exercise. Some found it was more convenient to track their activities with GPS because the application automatically measured detailed information about speed and distance and showed real-time data. However, they reported that they often forgot to start the GPS tracking.

### Intensity and satisfaction

Participants highlighted the importance of reporting the intensity of their activities afterwards: they found a big difference between having a training session or a match and doing an exercise just for fun. The intensity also had to be taken into account when calculating the points that could be earned. *“You should fill out the intensity…; when I am following a spinning lesson, then a specific amount of points are rated for that activity, but I can exercise to the maximum or I can exercise at ease” (male who met PA guidelines*).

Participants who did not meet PA guidelines wanted to be able to add information after completing their activities, such as how they felt during the activity. *“Maybe when you have finished running, you could indicate with a smiley how you felt during the exercise” (female who did not meet PA guidelines).*

### Extra device

A couple of participants wanted to use the app in combination with another device, such as a pedometer or heart rate monitor. Most who mentioned this functionality were already physically active. Others found it unnecessary and were not willing to pay extra for such a device.

### Coaching aspects

#### Coach

A coaching feature generally was seen as a huge advantage in a PA smartphone app. Some participants preferred the attention from a live personal coach or the support of friends during their activities. However, they recognised that if this was not possible, a coaching feature in an app is the second-best option. Opinions differed as to whether this coaching feature should provide support during or after PA. Some said they would find it annoying to hear a coach during their activities, primarily because they felt a device should not speak to them. However, most preferred to hear a motivating and enthusiastic voice giving information about their speed, distance or progress and making encouraging statements during PA. *“A coach who really encourages you, who is saying that you are doing a good job and who tells you to see you the next time, that is really nice” (female who did not meet PA guidelines).*

All participants liked the idea of a coaching feature, but depending on whether they met PA guidelines, they wanted it presented in a slightly different way. Those who did not meet PA guidelines wanted a coaching feature that would stimulate them to reach their goals, encourage them to keep going and provide them with tips about healthy exercising. Those who met PA guidelines wanted a coaching feature that would give detailed information about their workouts and tips on how to intensify the exercise as well as information about sporting events in the neighbourhood.

### Goal-setting

Almost all participants preferred a coach in combination with goal-setting. Most preferred to set goals when using the app. They wanted to choose between different goals or to be able to make a new goal, such as losing weight, improving fitness or keeping up with a specific activity schedule. They highlighted that if they could set a goal, they wanted the app to work as a coach by reminding them to exercise or to tell them what their progress was. It was very important to them to make a schedule, to set a task and to work toward reaching goals. Those who did not meet PA guidelines, in particular, preferred a goal-setting feature. They highlighted that they really needed to set goals and to be guided in reaching these goals. *“It is very important for me to set goals… with a graphic representation, like a bar, for example, you have a guideline to exercise a specific amount of hours per week, then it would be very good to see, ‘oh right now I am in the red zone or the orange zone,’ and when I am progressing, ‘I am in the green zone’” (female who did not meet PA guidelines).* Those who met PA the guidelines reported that goal-setting was unnecessary.

### Feedback and motivational features

Most participants would have liked to have some personal feedback from a coach after completing their activities. Examples of such personal feedback included compliments, reporting their progress and helping them with their schedule and reaching their goals. Adding tips to the app about how to reach goals, how to make activities more fun, how to exercise safely and when it is best to exercise would be desirable assets, according to the participants.

In addition, most reported a desire to add more information about themselves before using the app, such as their motivation level, experience level, desired goals and weights and heights. *“Maybe you could first fill out something about yourself, for instance how motivated you are and whether you are feeling good at the moment” (female who did not meet PA guidelines).*

In addition, they wanted to receive more detailed information about their activities afterwards. For instance, graphic visualisations of their progress, burned calories, a map of the route taken and speed and distance information. The group of participants who did not meet PA guidelines, in particular, preferred information about the number of calories burned during a workout. Information about the environment, such as operating hours of sports facilities, was identified as less important because they already knew it or could search for that type of information on the Internet. Opinions as to whether the app should offer information about the weather were diverse: for some, it would be helpful if the app could take the weather forecast into account when scheduling activities, but for others, it made the application less clear, and they could use the Internet just as easily. Some stressed that information about sporting events in their neighbourhood was appreciated.

Some participants suggested a music feature during their activities that could be interrupted by the coach. When doing a good job, this music feature could reward them with a ‘power song’ , motivating them to keep going.

### Competition

Most participants found the ranking feature interesting and motivating. They experienced this ranking as a match in which they did not want to be inferior to their friends. *“Yes it is a little bit shocking when you noticed that your friends did a good job” (female who did not meet PA guidelines). “Haha, I would go for a workout because it is confronting and because I want to be physically active…” (female who did not meet PA guidelines). “Yes, it encourages me. A friend of mine is jogging quite often, so when I see she did some exercise, it motivates me to go exercising again” (female who did not meet PA guidelines).*

A few participants reported the ranking feature as unimportant and something they did not need. They found it only interesting to compare their results with themselves and not with others. Additionally, because of their lack of time, they wanted to focus only on exercising and not on playing a game. Some participants who did not meet PA guidelines found it confrontational, leading to either a decrease or increase in PA.

A couple participants intended to continue using the app after this study. Their reasons were that (1) they started a competition with their friends that they wanted to continue or (2) they used the app to document their exercise progress.

### Rewards

Most participants liked earning points according to their exercise. Receiving an award was perceived as motivational and as input for a competition with friends. *“But it motivates me to log my activity, if you are going to the next level when you are filling in your activities. …Yes, I like that” (male who met PA guidelines*).

For some of the participants, it was unnecessary to get rewarded with points for being physically active. *“Yes, it doesn’t mean anything to me.” (female who met PA guidelines).*

What bothered most participants was that if they were rewarded with points, it was unclear how these points were calculated. They preferred that the number of points represent the type and intensity of the activity. Most wanted to receive real rewards instead of virtual rewards, such as discount vouchers for sporting goods stores, gift cards or tickets to sporting events. Some wanted to earn points that reflected their burned calories.

### Chat

Participants were clear about whether the app should have a chat feature. *“The idea is okay, but nobody uses it, so, yes, you don’t need it” (female who did not meet PA guidelines).* They were unanimous that the chat function was a needless feature and a waste of time. They highlighted that if they wanted to chat, they would use other apps.

### Sharing through social media

#### Reason for sharing through social media

Some participants reported that they occasionally shared their PA achievements through social media (primarily Facebook). The main reasons for sharing their results were that they were proud of their accomplishments or that they wanted to share information about, for example, their running or cycling routes with friends. The perceptions of their feelings if posts were liked or responded to were diverse. Some reported that it would motivate or support them, while others reported that it would not make a difference. Those who did not meet PA guidelines, in particular, acknowledged that they liked getting Facebook likes for their achievements, and they stated it would make a difference in their PA behaviours.

If participants shared their achievements through social media, they preferred doing so with personally typed messages, maps of their routes or photos. They also highlighted that sharing their achievements via the app seemed unnecessary, because they could share it via Facebook themselves.

Though some participants shared some of their achievements through social media using other smartphone apps, almost nobody shared them via the *Nexercise* app. In each focus group discussion, there was strong agreement that people should post only exceptional results, such as winning a match, becoming a champion, participating in a marathon or reaching a desired goal. The main reason for this preference was their annoyance at people who post all types of information (e.g., training results or walking to the bus stop), and they did not want to be perceived as that type of person. *“Yes indeed, why do others need to know,… it is like, oh I did some sports… It is a little bit stupid. That’s when I think to myself, nobody needs to know…” (female who did not meeting PA guidelines*).

Other reasons included being physically active for oneself, being embarrassed by the results, feeling it was not worth mentioning and feeling it was just as easy to tell friends that type of information in person.

### Private community

Many participants reported that they found most posts of others in their social media communities as annoying and something they were not interested in. They highlighted that this feeling depended on who shared the information (e.g., close friends or training buddies). They also reported that information shared by others about an exceptional accomplishment or reaching a goal was seen as something interesting to read. Therefore, in almost every focus group discussion, sharing achievements in a private community through social media was raised. Almost all participants reacted quite positively to the idea, and they were willing to form such a group with their closest friends, friends who were interested, people with the same goals, people using the same application, people with the same fitness level, people from the same sports club or people from their area of study. They envisioned that they would receive social support when part of a community with similar interests.

## Discussion

This study explored the use and appreciation of and the preferences for various features of a PA app by Dutch students (aged 18–25 years). As expected, based on the popularity of health and fitness apps, participants expressed positive attitudes toward a PA app. In general, they liked the idea of a PA app. Those who met PA guidelines thought that it was more useful to others than to themselves, stating that PA apps such as *Nexercise* could raise awareness for those who are not physically active, but that they are not suitable for themselves. Those who did not meet PA guidelines highlighted a desire for a personal coach function to help them achieve their self-determined goals, whereas those who met the guidelines preferred detailed training information, such as how to intensify their training sessions. Almost all participants preferred a companion website that could give detailed and general information about their behaviours.

The preferences for motivational features agree with those found in previous research; participants preferred self-monitoring and goal-setting features. Ehlers and Huberty [24] note that middle-aged women (mean age, 40.7 years; SD, 10.3 years) prefer a smartphone app that includes features to track their behaviour and to set goals; however, these women are less interested in motivational features or features to overcome barriers. Rabin and Bock [25] report similar results based on their study of fourteen adults (aged 23–60 years) who used three PA apps and felt that the ideal app should apply to different types of activities, be easy to use, track activity automatically and set goals. Features that target self-regulatory principles (e.g., self-monitoring, goal setting, behavioural feedback and problem solving features to overcome barriers) have been used successfully in PA promotion interventions. King and colleagues [26] demonstrate in a small study population that an app using self-regulatory principles is able to increase overall moderate-vigorous PA in aging adults. Kirwan and colleagues [13]. These results suggest that a PA app that uses self-regulatory principles could successfully increase PA in young adults.

Although 98% of Dutch young adults (aged 18–25 years) actively use social media [7], our study participants were not willing to share all their accomplishments on Facebook, suggesting that linking to social networking sites should not be a primary feature in PA app interventions. These results agree with those of Cavallo and colleagues [27], who conclude that their intervention among students aiming to increase social support for PA with online social networking did not improve perceptions of social support. Our participants reported that Facebook is not an appropriate platform to share their achievements because everybody is able to read their status updates. A private community could offer the possibility of sharing goals and achievements with peers. Further research is needed to explore whether such private social media communities could enhance social support, therefore enhancing the effects of PA apps in young adults.

### Implications for future interventions

This study suggests the need for an app in the form of a virtual coach to guide users who do not meet PA guidelines and to help them to overcome barriers, reach self-determined goals or monitor their progress. Feedback that is normally provided face-to-face by a personal coach should be integrated into the virtual coach. Besides the personalised and tailored feedback, the feedback should be rated as credible and trustworthy. Translating face-to-face feedback into a virtual coach requires a highly detailed diagnostic assessment for translating the information in a series of “if- then” messages that are linked to feedback messages and techniques for increasing PA.

In addition to an initial diagnostic assessment, the participants preferred ongoing assessments to adjust the feedback messages over time. The initial diagnostic assessment should be based on self-reported data to assess PA level, to identify barriers and to assess daily emotions. However, it should also be based on objective measures to assess the behaviours using GPS and/or an accelerometer. For future interventions, researchers and programmers will be challenged to build an appealing and engaging app that includes a diagnostic assessment able to gain detailed information with minimal burden on the participant and that will be used over a long period of time. However, because the majority of participants perceived the app to be enjoyable for a short period of time, more research is needed to examine whether a PA app alone is a promising tool for achieving long-term behaviour change or if it should be combined with other channels, such as a face-to-face programme. All participants identified features that would enhance the attractiveness of a PA app, such as self-monitoring features, competition features and goal-setting features. Competition may have been less-preferred by those who did not meet PA guidelines because it was perceived as confrontational by some. Therefore, when intervention designers add a competition feature, they should consider who would participate in the competition, so that the competition will be motivational and not frustrating.

### Strengths and limitations

A strength of this qualitative study is its ability to explore the students’ opinions, beliefs and experiences regarding PA apps. To our knowledge, this was the first to explore students’ appreciations and preferences, and therefore provides valuable information for future app-based interventions.

A limitation, also related to its qualitative explorative character, is that findings cannot be generalised, certainly not beyond the population of Dutch university students. To increase the generalizability to the Dutch young adult population, research should examine these appreciations and preferences among young adults in other groups within this age range. Furthermore, quantitative observational research and interventional studies in larger samples of young adults should be conducted to test our findings, including objectively measuring app usage. This study included a small sample size because of a high drop-out rate (47.4%) which may have created selection bias. A more representative sample may have led to different results, thus the app features we found to be desirable may not meet the needs for all potential app users. However, given that no new information was retrieved from the last focus group discussion, data saturation most likely was reached at least for the population of Dutch university students.

## Conclusions

In conclusion, this study provides explorative insights into the preferences of students regarding a PA app. Apps aiming to increase PA in young adults should provide personalised and tailored feedback and include a coaching function. A well-oriented and easy-to-use design must be developed, with the option to customise the application. Preferred features to be included in an application are ranking features, a coaching feature through which users are motivated during the exercise and receive feedback afterwards, and the possibility to set goals and to work with a schedule. In addition, participants prefer a website that accompanies the app to provide overviews of their results and progress. There is little need for a sharing feature to post results through social media.
